# Endotipsitis in an Immunocompetent Patient With Lactobacillus Bacteremia

**DOI:** 10.7759/cureus.33405

**Published:** 2023-01-05

**Authors:** Aleeya Shareef, Tahir Khan, Mohammad Shahid, Mustafa Musleh, Ehsan Shabbir

**Affiliations:** 1 Internal Medicine, Wright State University Boonshoft School of Medicine, Dayton, USA; 2 Internal Medicine, Premier Miami Valley Hospital, Dayton, USA; 3 Gastroenterology, Premier Miami Valley Hospital, Dayton, USA

**Keywords:** tips complications, infection of tips, lactobacillus, transjugular intrahepatic portosystemic shunt (tips), endotipsitis

## Abstract

Transjugular intrahepatic portosystemic shunt (TIPS) is a procedure commonly performed to decompress portal venous pressure since the early 1990s. Endotipsitis, which refers to persistent bacteremia caused by endovascular infection of the TIPS stent, is a rare but serious complication of this procedure. Very few cases of endotipsitis have been reported worldwide. We report the case of an immunocompetent patient diagnosed with endotipsitis, an atypical risk factor for *Lactobacillus* infection. This case report adds to the literature on underreported complications of TIPS, highlighting an urgent need for introducing clinical practice guidelines regarding the definition, diagnosis, and treatment of endotipsitis.

## Introduction

Transjugular intrahepatic portosystemic shunt (TIPS) is a procedure commonly employed for decompressing portal venous pressure. It is often used when other medical modalities fail to control variceal bleeding and ascites in patients with advanced liver disease [1]. Nevertheless, there are numerous additional indications for the TIPS procedure. Systemic complications related to TIPS include hepatic failure, encephalopathy, sepsis, and even death [2]. Local complications of the TIPS procedure include cardiac tamponade, laceration of vessels with resultant bleeding and pneumothorax, and endoprosthesis complications, such as migration, misplacement, and occlusion of the TIPS [2].

Fever in a patient with TIPS could be attributed to various causes, one of them being endotipsitis. Endotipsitis is a rare complication of the TIPS procedure that has been scarcely reported in the literature. Very few cases of endotipsitis have been described in the literature, and *Lactobacillus* bacteremia due to endotipsitis is extremely rare [1,3]. This report would be the fourth presumptive known case of *Lactobacillus* bacteremia associated with endotipsitis. The low incidence of this condition is primarily due to the lack of a universally accepted classification and underreporting. This report adds to the literature by presenting an immunocompetent patient with atypical signs associated with *Lactobacillus* bacteremia, resulting in a diagnosis of endotipsitis.

The abstract of this article was previously presented as a poster at the American College of Gastroenterology Conference in Charlotte, North Carolina on October 24, 2022.

## Case presentation

A 64-year-old female, with a medical history of nonalcoholic steatohepatitis, portal hypertension, cirrhosis, recurrent ascites, esophageal varices with bleeding, status-post (s/p) TIPS procedure nine months before the current admission, presented with chief complaints of fever, chills, somnolence, and progressive lethargy for the past three to four days. Upon chart review, the patient was found to have undergone the TIPS procedure due to continued variceal bleeding s/p banding and had been given vancomycin immediately prior to her procedure; however, further details and records were unable to be obtained since the procedure had been performed at an outside hospital.

Her vitals showed a temperature of 38.9 °C, heart rate of 144 beats per minute, blood pressure of 90/70 mmHg, and respiratory rate of 18 breaths per minute. Physical examination was normal aside from bilateral scleral icterus. There was no peripheral edema, asterixis, or abnormal heart/lung sounds. The patient's labs upon admission are presented in Table 1. Her urinalysis was normal.

**Table 1:** Laboratory values upon admission

Lab test	Patient value	Reference range
Hemoglobin (g/dl)	11.9	12.1–15.1
Hematocrit (%)	34.3	36–48%
White cell count (per mm^3^)	5	5–10
Segmented neutrophils (%)	93	47–55
Lymphocytes (%)	3	0.9–4.1
Platelet count (per mm^3^)	94	150–400
Sodium (mEq/L)	133	135–145
Potassium (mEq/L)	3.5	3.7–5.2
Blood urea (mg/dl)	15	6–20
Creatinine (mg/dl)	0.4	0.5–1.2
Calcium (mg/dL)	11.1	8.7–10.7
Blood glucose (mg/dL)	180	70–100
Ammonia (µ/dL)	40	15–45
Total bilirubin (mg/dl)	3.1	0.1–1.2
Direct bilirubin (mg/dl)	1	0.0–0.4
Alanine aminotransferase (IU/L)	62	4–36
Aspartate aminotransferase (IU/L)	120	8–33
Alkaline phosphatase (U/L)	114	20–130
Troponin (ng/mL)	324	0–0.04

**Figure 1:**
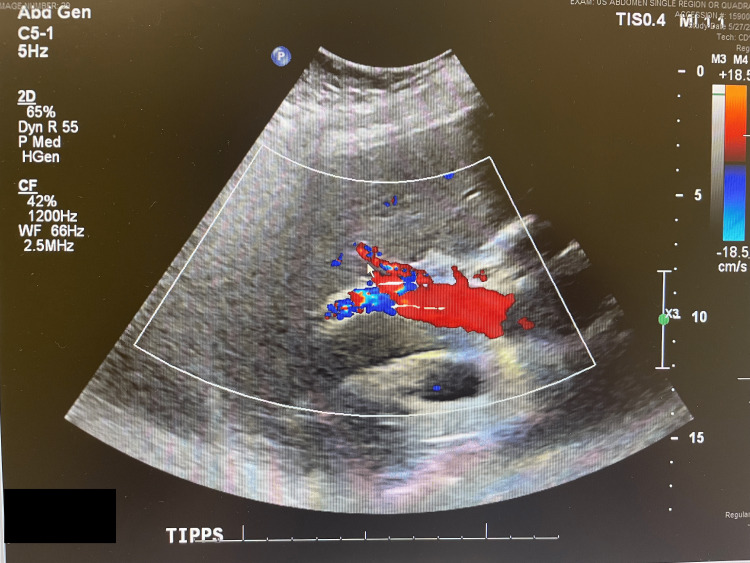
Patient's echocardiogram during her admission

Given the concern for a possible septic shock on presentation, a full infectious workup was performed, including chest X-ray, echocardiogram (Figure 1), right upper quadrant ultrasound, and CT of the abdomen and pelvis, revealing a patent TIPS. No vegetation, ascites, acute abnormalities, or obvious sources of infection were detected, as seen in Figure 1. Two separate blood cultures were obtained. Vancomycin and piperacillin-tazobactam were started.

On day one, blood cultures grew Gram-positive bacteria on both samples. The patient became afebrile on day two. Both samples grew *Lactobacilli*, which were pansensitive. Antibiotics were narrowed down to piperacillin-tazobactam. Repeat blood cultures on day three showed the clearance of *Lactobacilli* cultures. Gastroenterology recommended outpatient esophagogastroduodenoscopy (EGD) in two to three weeks to monitor the varices, as well as for screening purposes given the history of liver cirrhosis.

Given the patient's history of TIPS and no other source of infection, a “probable” diagnosis of endotipsitis was made. She was discharged with a 21-day course of intravenous (IV) piperacillin-tazobactam with a plan to repeat blood cultures after the treatment. If repeat blood cultures turned positive, a tagged white blood cell (WBC) scan and EGD were recommended. She completed the antibiotic course, and the cultures were found to be negative on day 28.

## Discussion

The initial differential diagnosis for our patient included septic shock, dehydration, gastroenteritis, hepatic failure, hepatic encephalopathy, pneumonia, and acute heart failure. Underlying diseases, such as endocarditis, abscess, bowel disease, and neoplasia, were either low on the differential diagnosis list or excluded. The investigation was thoroughly based on the obtained history regarding the duration of illness, the character of symptoms, localization, risk factors, and objective examination. Her laboratory evaluation was significant for anemia, thrombocytopenia, and hyperbilirubinemia, which could possibly be explained by her decompensated liver cirrhosis. However, laboratory results were also significant for hypercalcemia and an elevated troponin level, which subsequently down-trended during her admission. The patient denied any known sick contacts or recent history of traveling; therefore, tropical fevers or traveler's diarrhea/infection were deemed unlikely. The patient had a history of coronavirus disease 2019 (COVID-19) several months ago but was negative for COVID-19 upon admission. Chest X-ray ruled out any acute cardiopulmonary abnormalities, thereby excluding cardiologic or respiratory causes such as pneumonia or heart failure. Abdominal ultrasound demonstrated a patent TIPS and no ascites in the right upper quadrant, indicating that paracentesis was unlikely. A diagnosis of endotipsitis was made given the history of TIPS and with positive blood cultures being contributory for *Lactobacilli* growth, indicating bacteremia from an unknown source after obvious infective etiological causes were ruled out.

Sanya and Reddy coined the term endotipsitis, defining it as an infection of the TIPS. A “definite” infection was referred to as a “continuous, clinically significant bacteremia, with vegetations or thrombi inside the TIPS” [4]. Whereas a “probable” infection was described as “sustained bacteremia and unremitting fever in a patient with an apparently normal TIPS without an identified site of infection elsewhere in the body” [4]. Armstrong and MacLeod proposed another definition of endotipsitis: “a sustained bacteremia in a patient with a TIPS device, with or without thrombus, plus either no other identifiable infective focus or an identifiable infective focus that is not considered to be the source of the bacteremia after an exhaustive workup” [5].

However, the definitions of endotipsitis continue to be debated and a definitive definition has yet to be established. The lack of a universal definition often results in endotipsitis becoming a diagnosis of exclusion. Based on current definitions of endotipsitis and detailed examinations and analyses, our patient's condition was most closely compatible with the definition provided by Mizrahi et al., which states that “endotipsitis involves a persistent bacteremia and fever with either shunt occlusion, vegetation, or bacteremia in the presence of a patent shunt, and when other sources of bacteremia have been ruled out” [6].

The presentation of endotipsitis varies but almost always includes fever [5]. Other symptoms include malaise, chills, anorexia, diarrhea, and shock [5]. Several case reports have described associated jaundice attributed to sepsis, liver dysfunction along with hepatomegaly, frequent variceal bleeding, and recurrent ascites attributed to TIPS occlusion as presenting symptoms [6]. The pathogenesis suggests a damaged pseudo-endothelium involving the luminal surface of the stent graft [7]. As an endovascular infection, endotipsitis could be classified as an early infection if it occurs within 120 days of the TIPS procedure or a late endotipsitis infection when it manifests beyond this time frame [5]. A study from 2004 found that the average time for endotipsitis to occur was about 210 days (range: 6-732 days) [8]. Our patient was diagnosed with endotipsitis nine months after undergoing the TIPS procedure.

The most recent literature review from 2017 has reported 56 cases of endotipsitis; 32.1% of those cases resulted in death [6]. Enterococcus was the most common etiology of endotipsitis, with 14 cases reported and three resulting in death [4]. According to this review, there were three cases of endotipsitis complicated by *Lactobacilli* bacteria with two resulting in death [1]. This report is the fourth presumptive known case of *Lactobacilli* as an etiologic agent for endotipsitis. *Lactobacilli* are Gram-positive, catalase-negative, non-sporulating, aerobic or facultative anaerobic, rod-shaped bacteria [3]. They inhabit a wide variety of environments, including the gastrointestinal tract, oral cavity, and vagina, and have also been associated with fermented foods, such as probiotics [3]. However, our patient was not taking any probiotics, which excluded it as a risk factor. While *Lactobacillus* bacteremia accounts for only 0.1% of all positive blood cultures and is often described as contamination, the attributable mortality remains as high as 30% [9]. Despite the lack of risk factors for *Lactobacilli* bacteremia, it is likely that our patient was more susceptible to various infections due to the immune dysfunction that occurs with cirrhosis. 

Whenever endotipsitis is suspected, the workup should start with a color Doppler ultrasound to evaluate TIPS patency, along with the exclusion of all other possible sources of bacteremia [10]. An indium-111-labeled leukocyte (WBC) scan can also assist by demonstrating increased leukocyte uptake around the TIPS [11]. The gold standard method for the diagnosis of endotipsitis involves removing the TIPS and culturing it; however, this is not feasible without a concomitant liver transplant [12]. In one case review involving 36 endotipsitis patients, Doppler imaging demonstrated vegetation or thrombi only in 47% of patients [6].

Antibiotic therapy is recommended for approximately six weeks with repeat blood cultures to ensure early resolution of bacteremia [11]. In patients with continued bacteremia despite being on appropriate antibiotics, extraction of TIPS via liver transplant should be considered [13]. However, if an infection can be brought under control by antibiotics, a transplant may be curative, similar to patients with sclerosing cholangitis who undergo a liver transplant in the presence of active infection [4]. In our patient, we believe that bacteremia was not sustained due to early high suspicion of endotipsitis, an extensive infectious workup that was negative, and a prolonged course of piperacillin-tazobactam to treat this rare infection.

## Conclusions

Endotipsitis is a prosthetic infective complication that is uncommon but possibly life-threatening. It is difficult to establish a diagnosis of endotipsitis due to the lack of universal definitions and diagnostic criteria; therefore, physicians should maintain a high index of suspicion when a patient with TIPS is diagnosed with bacteremia. *Lactobacillus* bacteremia should not always be considered contamination, especially in patients with TIPS or those who are immunocompetent. Awareness among clinicians regarding this condition needs to be improved to avoid underdiagnosis. Further studies are required to standardize diagnostic and treatment algorithms because endotipsitis remains an uncommon infection with significantly high morbidity and mortality associated with it.
